# A MEMS Device Capable of Measuring Near-Field Thermal Radiation between Membranes

**DOI:** 10.3390/s130201998

**Published:** 2013-02-04

**Authors:** Chong Feng, Zhenan Tang, Jun Yu, Changyu Sun

**Affiliations:** School of Electronic Science and Technology, Faculty of Electronic Information and Electrical Engineering, Dalian University of Technology, Dalian 116023, China; E-Mails: amfengchong@gmail.com (C.F.); tangza@dlut.edu.cn (Z.T.); scy_1010@163.com (C.S.)

**Keywords:** MEMS, freestanding membrane, near-field, thermal radiation

## Abstract

For sensors constructed by freestanding membranes, when the gap between a freestanding membrane and the substrate or between membranes is at micron scale, the effects of near-field radiative heat transfer on the sensors' thermal performance should be considered during sensor design. The radiative heat flux is transferred from a membrane to a plane or from a membrane to a membrane. In the current study of the near-field thermal radiation, the scanning probe technology has difficulty in making a membrane separated at micron scale parallel to a plane or another membrane. A novel MEMS (micro electromechanical system) device was developed by sacrificial layer technique in this work to realize a double parallel freestanding membrane structure. Each freestanding membrane has a platinum thin-film resistor and the distance between the two membranes is 1 *μ*m. After evaluating the electrical and thermal characteristics of the lower freestanding membrane,experimental measurements of near-field radiative heat transfer between the lower membrane and the upper membrane were carried out by setting the lower membrane as a heat emitter and the upper membrane as a heat receiver. The near-field radiative heat transfer between the two membranes was validated by finding a larger-than-blackbody radiative heat transfer based on the experimental data.

## 1. Introduction

Freestanding micro-mechanical membrane structures have been developed and applied as a variety of sensors [1–10]. Measuring the temperature change of the freestanding membrane is the basic principle of these sensors. The thermal performance of these freestanding membrane structures are key factors affecting the sensitivity of these sensors. Thermal conduction and thermal radiation are two generally considered heat transfer modes of a freestanding membrane working in vacuum.

However, an proximity effect on thermal radiation was found by Domoto and Hargreveas in the late 1960s [11–14], which is called the near-field thermal radiation. The radiative heat power per unit temperature difference of the near-field radiation between two SiO_2_ (silicon oxide) planes has been found to be 6 nW and 18nW at the gap of 2.5 *μ*m and 30 nm, respectively [15]. They are higher than the 5.45 nW of the far-field radiation under the same temperature conditions. The distance between the freestanding membrane and the substrate or between two membrane is from micron to submicron scale for sensors fabricated by front-side surface micromachining techniques [16,17]. The near-field radiative heat transfer occurs at the micron or the submicron distance and brings away more heat from the freestanding membrane. The near-field radiative heat transfer mode needs to be studied to direct the structural design of the sensors.

Furthermore, for the freestanding membrane structure of the sensors, the radiative heat flux is transferred from the membrane and the substrate or between membranes. Despite the fact that the scanning probe technique has been successfully invented by some researchers to study the near-field thermal radiation between bulk materials [15,18–23], this technique is difficult to parallelize membranes separated at micron or submicron scale.

In this paper, a novel device with double freestanding membranes, named as DFM, was developed by MEMS (micro electro-mechanical system) process. The two membranes are parallel to each other and the distance between them were designed to be 1,000 nm implemented by aluminium sacrificial layer.Each membrane has a Pt (platinum) thin-film resistor so that it can be heated. The lower membrane of a DFM was firstly heated by supplying a series of constant currents under high vacuum condition. Then the upper membrane of the DFM was removed to realize a device with the lower freestanding membrane, named as SFM. The freestanding membrane of the SFM was heated to the same temperatures of the lower membrane of the DFM. Heating power differences between the two experiments were calculated from the measured data. The near-field radiative heat transfer between the lower membrane and the upper membrane of the DFM were calculated by the heating power differences. A larger-than-blackbody radiative heat transfer was found between the two membranes at the gap of 1,000 nm.

## 2. Design and Fabrication

### 2.1. Design

**Figure 1.**
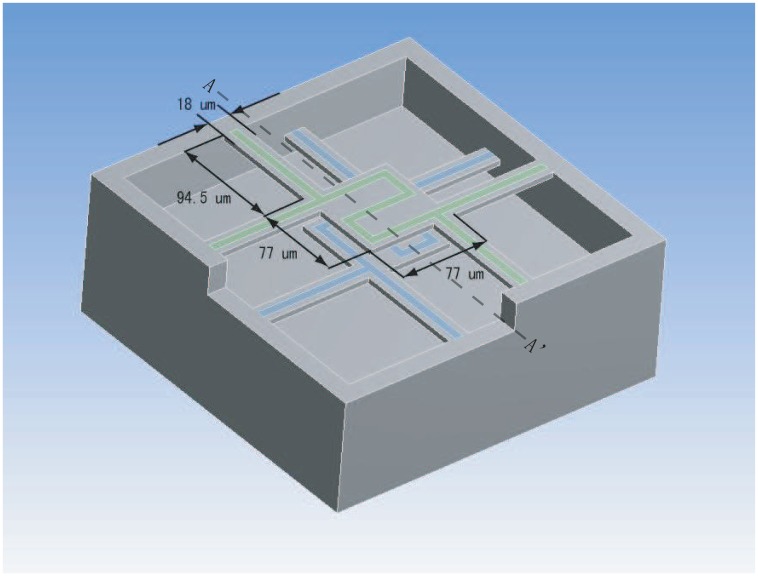
3D diagram of the sensor.

Figure 1 illustrates the structure of the device. The device consists of two freestanding membranes. Each freestanding membrane was made from a sandwich structure that included SiO_2_ (400 nm thick), SiN (200 nm thick) and SiO_2_ (200 nm thick). A Pt line of 7*μ*m width and 100nm thickness forms the resistor of each membrane, and the resistors work as both heater and thermometer. The Pt line has a zigzag shape and four ends so that it can uniformly provide joule heat over the membrane and precisely measure the average temperature of the freestanding membrane through the four-point method. Four beams support the four ends of the Pt resistor at two diagonal corners. The dimensions of a supporting beams were 94.5 *μ*m in length and 18 *μ*m in width.

A theoretical calculation of heat conductive coefficient of the four supporting beams was based on the dimensions of one supporting beam and thermal conductivity in Table 1. The total heat conductive coefficient is

(1) $$G_{\text{con}}=4\times \left(\lambda_{\text{SiN}}\frac{A_{\text{SiN}}}{L_{\text{SiN}}}+\lambda_{{\text{SiO}}_{2}}\frac{A_{{{\text{SiO}}_{2}}_{2}}}{L_{{{\text{SiO}}_{2}}_{2}}}+\lambda \text{pt}\frac{A_{\text{pt}}}{L_{pt}}\right)$$

where *A* is the cross-sectional area of the supporting beam, *L* is the length of the supporting leg and λ is thermal conductivity of material. The factor 4 in the formula means that each freestanding membrane structure has four supporting beams. The result was 2.89 × 10^−6^ W · K^−1^.

### 2.2. Fabrication

**Figure 2.**
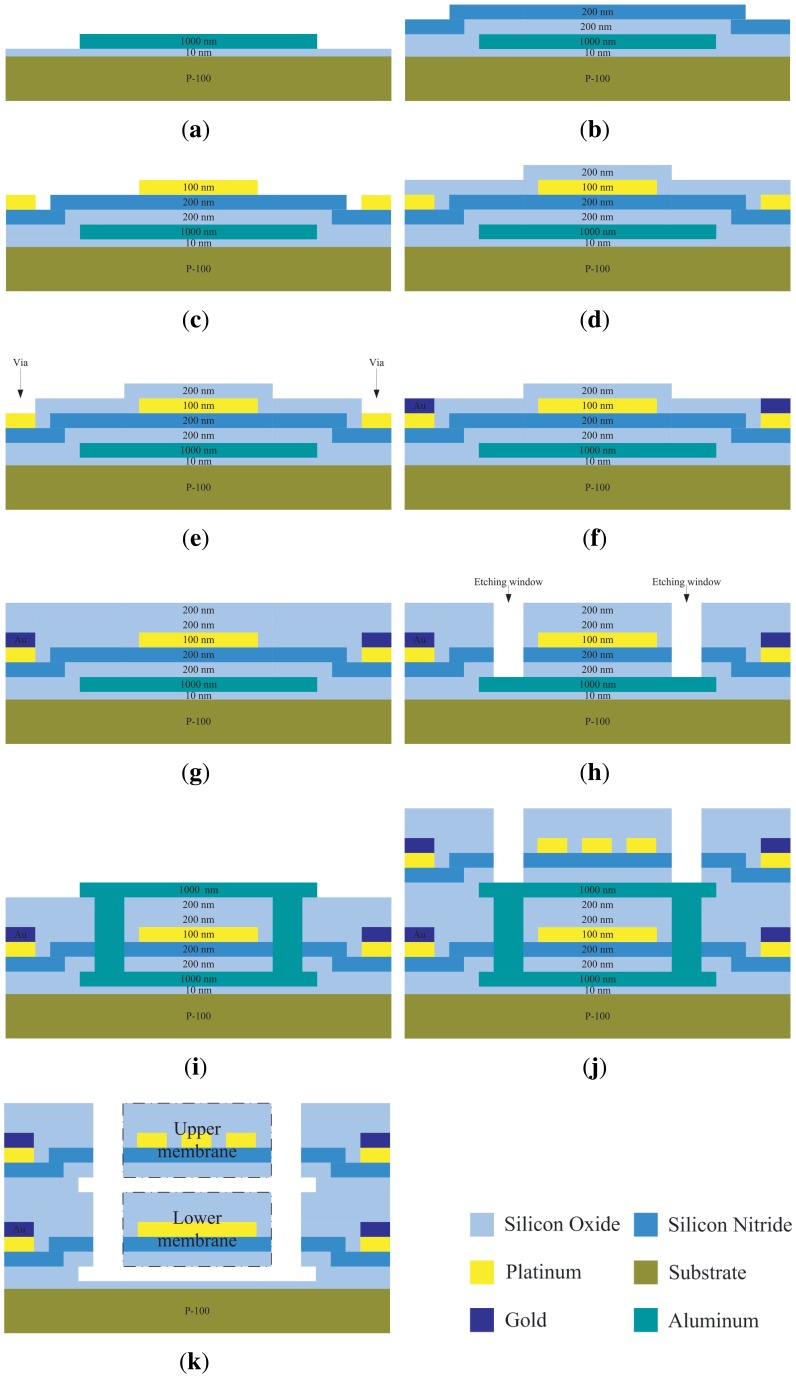
Process flow of a DFM fabrication. The cross section was at a–a′ in Figure 1.

The fabrication of a DFM had two processes: a MEMS process and a post-MEMS process.The MEMS process started with a 400 *μ*m thick 4″ silicon wafer as follows:

- A 1,000 nm thick aluminum was sputtered after 10 nm thick thermal oxidation SiO_2_ on the wafer (Figure 2(a)).
- A 200 nm thick SiO_2_ and a 200 nm thick SiN were sequentially deposited by PECVD (plasma-enhanced chemical-vapor) (Figure 2(b)).
- A 100 nm thick Platinum was sputtered and then patterned by lift-off technique (Figure 2(c)).
- A 200 nm thick SiO_2_ layer was deposited by PECVD (Figure 2(d)).
- The SiO_2_ layer in step d then was etched by RIE (reactive-ion etching) to open via (Figure 2(e)).
- A 200 nm thick gold internet line was sputtered and then patterned by lift-off technique (Figure 2(f)).
- A 200 nm thick SiO_2_ was deposited by PECVD (Figure 2(g)).
- RIE etching SiO_2_, SiN and SiO_2_ in the patterned regions until the aluminum layer was exposed (Figure 2(h)).
- A 1,000 nm thick aluminum was sputtered as the second sacrificial layer (Figure 2(i)).
- Repeat steps from (b) to (g) (Figure 2(j)).

As shown in Figure 2(h), the first freestanding membrane (also called the lower membrane) structure and its supporting beams were completed at the same time in step (g). The second freestanding membrane (also called the upper membrane) was finished after step (j), which has the same dimensions as the lower membrane.

**Figure 3.**
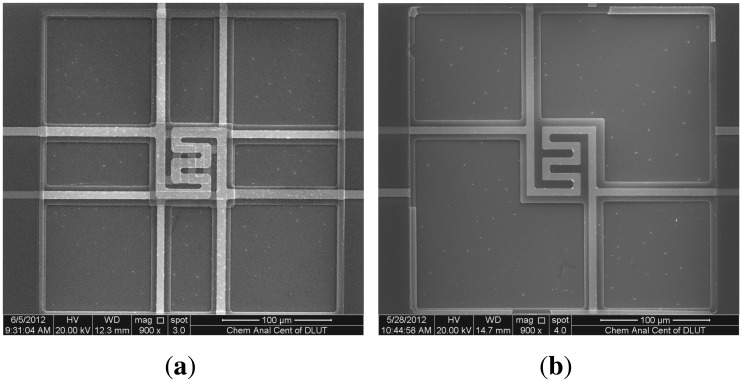
(**a**) SEM image of a DFM. (**b**) SEM image of a SFM.

The post-MEMS process was a wet etching process for releasing the two membranes of a DFM after wafer dicing. As seen in Figure 2(j), two aluminum layers were united so that it can be etched by one etching process. The etching solution is 85% Phosphoric acid and the temperature condition of the etching process is 80°C. By removing of the sacrificial Al layers, the two membranes are suspended, as shown in Figure 2(k). After an annealing process (350°C, 2 h) to release of residual stress of the membrane, a DFM had been completed. The SEM (scanning electron microscope) photograph of a DFM is shown in Figure 3(a).

As seen in Figure 3(b), the upper membrane of a DFM was removed by probe to fabricate a SFM after the post-MEMS, which certified that the two freestanding membranes were not adherent.

## 3. Measurements

### 3.1. Principle

Only the lower membrane of the device was used to measure the radiative heat transfer because the resistor in the upper one was not fabricated by incomplete via etching in the MEMS process. In the measurement, the lower membrane worked as a heat emitter and the upper membrane worked as a heat receiver. The heat balance equation of the emitter was similar to that of microbolometer. While a microbolometer is heated to a temperature *T* by an electric power *P* and does not absorb power from any external source of radiation in excess of that due to the surrounding at ambient temperature *T*_0_, its differential heat balance equation is described as [25]

(2) $$\text{C}\frac{dT}{dt}+(G_{\text{con}}+G_{fr})(T-T_{0})=P$$

where,

*t* is time, *C* is the thermal capacitance of the microbolometer,

G_con_ is the heat conductive coefficient determined by the dimensions of the supporting beam and the conductivity of the beam's materials,

G_fr_ is the far-field radiative heat transfer coefficient given by Stefan–Boltzmann law [26],

(3) $$G_{\text{fr}}=4\epsilon \delta AT^{3}$$

where,

*ϵ* is the total hemispherical emissivity,

*δ* = 5.67 × 10^−8^ W · m^−2^ · K^−4^ is Stefan–Boltzmann constant,

A is the radiation area of the microbolometer.

In the steady state we have

(4) $$(G_{\text{con}}+G_{fr})(T-T_{0})=P$$

For the emitter of the SFM, its steady heat balance equation is

(5) $$({\text{G}}_{\text{con}}+{\text{G}}_{\text{fr}})(T_{\text{sfm}}-T_{0})=P_{\text{sfm}}$$

Assuming that the temperature of the receiver is equal to the ambient temperature, the steady heat balance equation of the emitter of a DFM is:

(6) $$(G_{\text{con}}+G_{fr}+G_{nr})(T_{\text{dfm}}-T_{0})=P_{\text{dfm}}$$

where, the G_nr_ (*T_dfm_* – T_0_) is the near-field radiative heat transfer between the emitter and the receiver.Because the SFM was fabricated by the DFM, the emitter is same. The near-field heat transfer between the emitter and the receiver of the DFM can be calculated by the heating power difference between the DFM and the SFM while *T_dfm_* = *T_sfm_* = *T*, which is express as:

(7) $$(G_{nr}+T-T_{0})=P_{\text{dmf}}-P_{\text{smf}}$$

Finally, the near-field radiative heat transfer coefficient G_nr_ is

(8) $$G_{nr}=\frac{P_{\text{dmf}}-P_{\text{smf}}}{T-T_{0}}$$

### 3.2. TCR of the Pt Resistor

**Figure 4.**
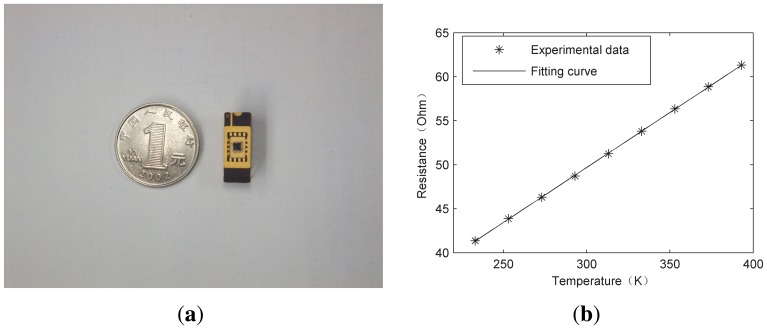
**(a)** a packaged device. (**b**) The TCR of the emitter Pt resistor.

The emitter's temperature *T* in Equation (8) was detected by the Pt resistor of the emitter.The relationship between resistance and temperature of the Pt resistor is described by the TCR (temperature resistance coefficient). After packaging the sensor, as shown in Figure 4(a), the TCR was experimentally determined in the vacuum chamber of a CCS-400H/204 close cycle refrigerator system (Janis Research Co., Wilmington, DC, USA). A KEITHLEY 2400 (Keithley Instruments, Inc., Cleveland, OH, USA) forced a constant driving current and measured the resistance with the four-point method. As shown in Figure 4(b), resistances of the Pt resistor of the emitter were recorded when the surrounding temperature changed from 233 K to 393 K at interval of 20 K. Fitting the measured data to a straight line, we had the relationship between the resistance *R_T_* and temperature *T* of the Pt resistor:

(9) $$R_{T}=0.12494T+12.207$$

Since the resistance is 46.3 and 58.86 Ohm at 273 and 373 K, respectively, the TCR (noted as *α*) is 2.7%.

### 3.3. Thermal Characteristics

In the radiative heat transfer experiments, the emitter cannot be operated above the maximum current that the emitter was able to withstand. The maximum current is given by a formula that has been presented in our previous work [27]:

(10) $$I\leq \sqrt{G_{0}{\left(dR_{T}/dT\right)}^{-1}}$$

where, *I* is the heating current, *dR_T_*/*dT* can be computed by Equation (9).

*G*_0_ includes G_con_ and G_fr_. Although the theoretical value of G_con_ was 2.89 × 10^−6^W·K^−1^ (see Section 2.1), its actual value cannot completely rely on theoretical calculation because of the thermal conductivity difference between bulk and thin film, and of the differences between the design and the sensor manufacturing. We chose a thermal time delay method to evaluate the actual value of heat conduction, the maximum heating current and the thermal stabilization time.

The emitter of SFM was used in the heating experiment of the thermal time delay method. A series of square wave currents were applied on the Pt resistor to heat the emitter. The frequency and the ratio of the square wave is 5 Hz and 50%, respectively. The amplitude of the square wave was set up based on Equation (9) and the theoretical value of G_con_. It was found that the emitter can be heated to 313 K by a 1.062 mA current while the ambient temperature is 293 K. For comparison, we chose heating currents as 0.8, 0.9, 1.0 and 1.1mA.

**Figure 5.**
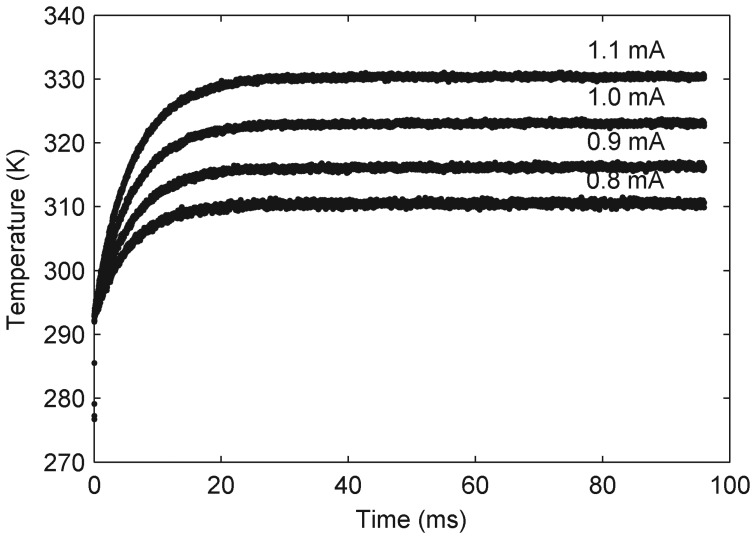
Temperature response curves of the emitter in the thermal time delay experiment.

The experiment was carried out under vacuum conditions (10^−6^ mbar) while the ambient temperature was 293 K. The chosen currents were supplied by KEITHLEY 2400 and voltages of the Pt resistor were recorded by DPO 3052 (Tektronix, Inc.). Resistances of the Pt resistor according to the heating currents were obtained by dividing the voltages with the currents. Temperatures of the emitter along with time were calculated by the resistances and Equation (9), as shown in Figure 5. Steady temperatures according to each heating current are listed in Table 2. A temperature rise of 46 K is observed when the heating current is 1.1 mA.

The G_con_ of the supporting beams can be obtained by fitting the experimental data curves with the temperature dynamic behavior of the emitter. Replacing *P* by a heating power *I*^2^ · *R* and momentarily ignoring the G_fr_ in Equation (2), we have

(11) $$\text{C}\frac{\text{dT}}{\text{d}t}+{\text{G}}_{\text{con}}(T-T_{0})=I^{2}R$$

By solving Equation (11), we can obtain the temperature dynamic behavior of the emitter as

(12) $$T=T_{0}+\frac{I_{2}R_{T0}}{\left({\text{G}}_{\text{con}}-I^{2}\alpha R_{T0}\right)}\left(1-\exp[-\frac{\left({\text{G}}_{\text{con}}-I^{2}\alpha R_{T0}\right)}{C}t]\right)$$

where *α* is the TCR of the Pt resistor.

Fitting the experimental data by Equation (12), we got G_con_ that included the G_fr_. The fitting results are listed in Table 2. G_fr_ were also listed in Table 2, which were calculated by Equation (3) while the *ε* is 0.35 [15] and A is 77 × 77 *μ*m^2^ as seen in Figure 1. As G_con_ is about 100 times of G_fr_, G_fr_ can be ignored in Equation (2) practically.

Therefore, the average value of G_con_ was 1.325 × 10^−6^ W·K^−1^, and by substituting the G_con_ into Equation (10), the maximum heating current was 3.26 mA. The radiative heat transfer measurement had to be done at the thermal steady state of the emitter according to Equation 7. The emitter thermal stabilization time was 30 ms, which was obtained from the fitting result also.

### 3.4. Measuring Near-Field Radiative Heat Transfer

Based on the principle in Section 3.1, experiments of measuring the near-field radiative heat transfer includes the following steps :

- The emitter of a DFM was heated by a series of constant currents (from 0.8 mA to 1.8 mA);
- The receiver of the DFM was removed by a probe to make a SFM;
- The emitter of the SFM was heated by a series of constant currents (from 0.8 mA to 1.6 mA).

**Figure 6.**
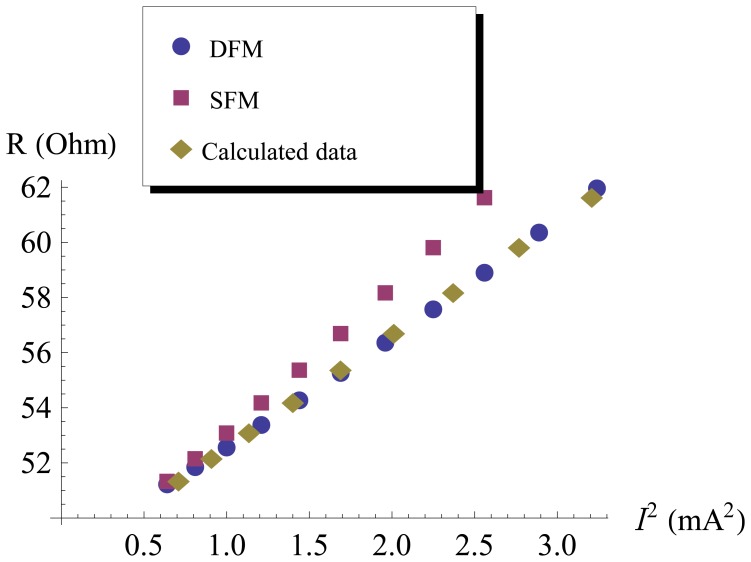
Resistances of the emitter of the DFM and the SFM corresponding to the heating currents.

The experiments were carried out in vacuum (10^−6^ mbar) at ambient temperature (293 K). All heating currents were supplied by KEITHLEY 2400, all resistances of the Pt resistor corresponding to the heating currents were recorded by KEITHLEY 2400 in the four-point method and the sampling delay time was 1 s. The experimental data are shown in Figure 6.

## 4. Results and Discussion

The near-field thermal radiation between the emitter and the receiver were calculated by the experimental data. Firstly, the functional relationship of the resistance and the square of heating current of the DFM were fitted as

(13) $$I^{2}=-11.7712+0.24288R$$

**Figure 7.**
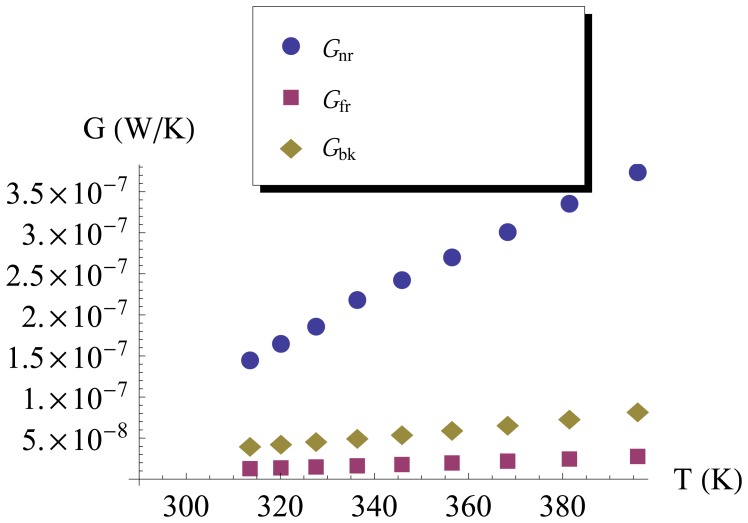
Temperature dependent heat transfer coefficient for far-field thermal radiation, blackbody radiation and experimental data.

By substituting the resistance of the SFM into Equation (13), the heating currents $\left(I_{\text{cal}}^{2}\right)$ were then estimated, which were depicted by diamond points in Figure 6. Next, each heating power difference at the same emitter temperature between the DFM and the SFM was evaluated by $\left(I_{\text{cal}}^{2}-I_{\text{sfm}}^{2}\right)R$, and the temperature of the emitter was evaluated by the resistance and TCR of the Pt resistor. Finally,the near-field radiative coefficient G_nr_ was calculated by Equation (8), which was shown in Figure 7.For comparison, a black body radiative coefficient G_bk_ was shown in Figure 7 also, which was calculated by Equation (3) while the *ε* is 1.

As shown in Figure 7, all G_nr_, G_fr_ and G_bk_ increase as the temperature increases, and G_nr_ exceeded the G_fr_ and G_bk_. The fact that G_nr_ is larger than G_bk_ indicates that the radiative heat power is higher than that of black body. Some researchers had reported that there are two modes of the thermally excited electromagnetic waves [28–30]: one is the propagating mode that can leave the surface of the emitter and radiate freely into the space; another is the evanescent mode (called surface electromagnetic waves also) that propagates along the surface and decreases exponentially in the perpendicular direction. The upper limit of the propagating mode electromagnetic waves' contribution to the radiative heat power is governed by Planck's law of black body [31]. The surface electromagnetic waves can contribute to the radiative heat transfer when a second surface is brought close to the first to enable photon tunneling. The radiative heat power can exceed that of black body. Therefore, the photon tunneling phenomenon happened between the upper membrane and the lower membrane at the gap of 1 *μ*m.

The near-field radiative heat transfer at 1 *μ*m distances will affect the thermal performance of the freestanding membrane. G_nr_ at 313 K was 1.46 × 10^−7^ W·K^−1^, about 10 times of G_fr_; G_nr_ at 396 K was 3.75 × 10^−7^ W · K^−1^, about 12 times of G_fr_. The heat conductive coefficient G_con_ of the emitter is 1.325 × 10^−6^ W · K^−1^ as measured in Section 3.3. When the temperature is 313 K, G_nr_ is 8% of G_con_. When the temperature is 396 K, G_nr_ is 28% of G_con_. Thus, at a higher temperature, the near-field thermal radiation has greater impact on the thermal performance of the emitter. Furthermore, the near-field radiative heat transfer should be taken into account to improve the sensitivity of those sensors that have freestanding membrane at a distance of 1 *μ*m to the substrate [3,32].

## 5. Conclusions

A novel device named DFM that has two plane-plane parallel membranes has been successfully implemented by the surface manufacturing technology. Each membrane has a Pt resistor with TCR of 2.7 %. For the lower membrane, a 46 K temperature rise corresponding to a 1.1 mA current was observed, indicating that the lower membrane is completely released. The measured conductive coefficient of SFM was 1.325 × 10^−6^ W·K^−1^. The heating power was different between DFM and SFM (the same device after removing the upper membrane) when heating the lower membrane to the same temperature. The near-field radiative heat transfer coefficients G_nr_ in response to temperatures were evaluated based on the heating power differences. Results show that G_nr_ is about ten times larger than black body radiative coefficient Gbk in the temperature range of 300–400 K. The experimental data can direct engineers to properly evaluate the effect of the near-field thermal radiation at 1 *μ*m distance on their sensors. The current technology should be improved to allow the detection of the actual temperature of the upper freestanding membrane.

## Figures and Tables

**Table 1.** Material thermal property [24].

	Platinum	Silicon oxide	Silicon nitride
Thermal conductivity λ (W · m^−1^ · K^−1^)	71.6	1.17	3.2

**Table 2.** Fitting result of thermal time delay experiment.

Heating current of the emitter I (mA)	0.8	0.9	1.0	1.1
Steady temperature T (K)	316.3087	323.0872	330.387	339.1644
G_con_ (W·K^−1^)	1.34 × 10^−6^	1.33 × 10^−6^	1.32 × 10^−6^	1.31 × 10^−6^
G_fr_ (W·K^−1^)	1.49 × 10^−8^	1.59 × 10^−8^	1.70 × 10^−8^	1.84 × 10^−8^
